# Bio F_1_B hamster: a unique animal model with reduced lipoprotein lipase activity to investigate nutrient mediated regulation of lipoprotein metabolism

**DOI:** 10.1186/1743-7075-4-27

**Published:** 2007-12-10

**Authors:** Sukhinder Kaur Cheema, Marion L Cornish

**Affiliations:** 1 Department of Biochemistry, Memorial University, St. John's, NL, A1B 3X9, Canada

## Abstract

**Background:**

Bio F_1_B hamster is an inbred hybrid strain that is highly susceptible to diet-induced atherosclerosis. We previously reported that feeding a high fat fish oil diet to Bio F_1_B hamster caused severe hyperlipidaemia. In this study we compared the effects of various diets in the Bio F_1_B hamster and the Golden Syrian hamster, which is an outbred hamster strain to investigate whether genetic background plays an important role in dietary fat mediated regulation of lipoprotein metabolism. We further investigated the mechanisms behind diet-induced hyperlipidaemia in F_1_B hamster.

**Methods:**

The Bio F_1_B and Golden Syrian hamsters, 8 weeks old, were fed high fat diets rich in either monounsaturated fatty acids, an n-6: n-3 ratio of 5 or a fish oil diet for 4 weeks. Animals were fasted overnight and blood and tissue samples were collected. Plasma was fractionated into various lipoprotein fractions and assayed for triacylglycerol and cholesterol concentrations. Plasma lipoprotein lipase activity was measured using radioisotope method. Microsomal triglyceride transfer protein activity was measured in the liver and intestine. Plasma apolipoproteinB48, -B100 and apolipoprotein E was measured using Western blots. Two-way ANOVA was used to determine the effect of diet type and animal strain.

**Results:**

The fish oil fed F_1_B hamsters showed milky plasma after a 14-hour fast. Fish oil feeding caused accumulation of apolipoproteinB48 containing lipoprotein particles suggesting hindrance of triglyceride-rich lipoprotein clearance. There was no significant effect of diet or strain on hepatic or intestinal microsomal triglyceride transfer protein activity indicating that hyperlipidaemia is not due to an increase in the assembly or secretion of lipoprotein particles. F_1_B hamsters showed significantly reduced levels of lipoprotein lipase activity, which was inhibited by fish oil feeding.

**Conclusion:**

Evidence is presented for the first time that alterations in lipoprotein lipase activity and mRNA levels contribute to varied response of these hamsters to dietary fat, highlighting the importance of genetic background in the regulation of lipid and lipoprotein metabolism by dietary fats. Bio F_1_B hamster may prove to be an important animal model to investigate nutrient mediated regulation of metabolic parameters under lipoprotein lipase deficiency.

## Background

High levels of plasma triglycerides (TG) are considered to be an independent risk factor for the development of cardiovascular disease (CVD) [1]. Hypertriglyceridaemia is representative of abnormal postprandial lipemia, and is defined as elevated postprandial TG concentrations above 2.26 mmol/L, and commonly accompanies the development of Type II Diabetes and obesity [2]. Hypertriglyceridaemia arises mainly from alterations in lipid and lipoprotein metabolism that affect the clearance of TG-rich lipoproteins, namely very-low density lipoproteins (VLDL) and chylomicrons. This increase can be due to several factors; i.e. increased production and secretion of VLDL and chylomicrons from the liver and intestine respectively, a decrease in their hydrolysis by lipoprotein lipase (LPL) or hepatic uptake by the low-density lipoprotein (LDL) receptor. The subsequent development of hypertriglyceridaemia is detrimental to cardiovascular health as it promotes a decrease in high-density lipoprotein (HDL) concentrations, a simultaneous increase in LDL, and an increase in the prevalence of small, dense LDL particles. These factors are characteristic of an atherogenic lipoprotein phenotype that increases the risk for the onset of CVD [3,4].

Dietary fat intake has the most influence on both the development and demise of hypertriglyceridaemia. Saturated fatty acids (SFA) are known to increase LDL-cholesterol and promote the formation of atherosclerotic lesions, while unsaturated fatty acids, particularly omega-3 (n-3) fatty acids are known to decrease TG concentrations [5]. The marine derived n-3 fatty acids; eicosapentaenoic acid and docosahexaenoic acid, found in fish oil, are suggested to be one of the most successful dietary interventions for reducing hypertriglyceridaemia [6]. These fatty acids have been shown to maintain a positive lipoprotein phenotype by decreasing plasma TG concentrations, decreasing VLDL synthesis and secretion, decreasing the production of apolipoproteinB100 (apoB100), and increasing the clearance of TG-rich lipoproteins [7]. However, the effect of fish oil on LDL levels and lipoprotein lipase activity is less consistent. Fish oil has been shown to increase, decrease, or have no effect on plasma LDL-cholesterol [8,9], and LPL activity [10-13].

We previously used F_1_B hamster to investigate the regulation of lipid and lipoprotein metabolism by fish oil [14]. Surprisingly, we found that high levels of fish oil induced severe hyperlipidaemia in F_1_B hamsters. The presence of severe hyperlipidaemia in fish oil fed F_1_B hamsters indicated either an enhanced synthesis of TG-rich lipoproteins or inhibited clearance of TG-rich lipoproteins. In this study, we compared the effects of various diets, including high fat fish oil diet, in the Bio F_1_B hamster and the Golden Syrian (GS) hamster. We further investigated the mechanisms behind hyperlipidaemia in the F1B hamsters. The Bio F1B is derived from two highly inbred lines, by crossbreeding between Bio 87.20 female with a Bio 1.5 male. The Bio F1B hamster was initially used as a genetic defined control for cardiomyopathy. However, it has recently become an exciting animal model for hyperlipidemic-related applications. The Golden Syrian hamster, on the other hand, is an outbred hamster strain which is used as a control. Our findings highlight the importance of genetic background in regulating plasma lipid and lipoprotein levels.

## Methods

### Animals and Diets

Seven week old, male Bio F_1_B and Golden Syrian (GS) hamsters were obtained from Biobreeders Inc. (Massachusetts, USA) and Charles River Labs (Kingston, NY, USA) respectively. The animals were kept on a chow diet for one week prior to feeding the specified diets. After this equilibration period, animals in each strain were divided into one of three groups (n = 12, F_1_B; n = 12, GS) and fed one of the three specified diets for a period of four weeks. These specified diets consisted of fat-free semi-purified diet (ICN biomedicals, Ohio, USA) supplemented with 200 g/kg (20% w/w) of either fish oil (FO) (Menhaden oil, Sigma-Aldrich, Ontario, Canada), high monounsaturated fatty acid (MUFA) safflower oil obtained from a local supermarket (MUFA), or a diet designed to give the animals an n-6:n-3 fatty acid ratio of ~5 (n6:n3). Due to the presence of 2.5 g/kg cholesterol in the fish oil diet, cholesterol was added to the MUFA and n6:n3 diets in appropriate proportions. The diets were kept at -20°C and fresh diets were given daily *ad libitum*. The composition of all three experimental diets is given in Table 1. Lipids were extracted from all diets and analyzed via gas liquid chromatography [15], the fatty acid composition of which is given in Table 2. Food intake was measured daily and animal body weight was measured weekly. There was no significant effect of diet on food intake or body weight in F_1_B and GS hamsters (data not shown). The animals were housed in individual cages in a single room. The room was lit from 0700 hrs to 1900 hrs with the temperature maintained at 21°C and humidity at 35 ± 5%. All procedures were approved by Memorial University's Institutional Animal Care Committee in accordance with the guidelines of the Canadian Council for Animal Care.

**Table 1:** Nutrient composition of the high fat* semi-purified diets^† ^(g/kg)

Diet	Fish Oil	MUFA	N6:N3
Casein	200	200	200
DL-Methionine	3	3	3
Sucrose	305	305	305
Maize Starch	190	190	190
Vitamin mix^‡^	11	11	11
Mineral mix^‡^	40	40	40
Fibre^§^	50	50	50
Fat	200	200	200
Cholesterol	2.5	2.5	2.5

* Semi-purified diet designed for a 200 g/kg fat level.

^† ^Diet ingredients from ICN Biomedicals, Aurora, OH, USA.

^‡^Supplied in quantities adequate to meet nutritional requirements (National Research Council, 1995).

^§ ^Supplied as Alphacel non-nutritive bulk (ICN Biomedicals, Aurora, OH, USA). MUFA, monounsaturated fatty acid rich diet

**Table 2:** Fatty acid composition of various diets

Fatty Acid	Diet		
(%)	FO	MUFA	N6:N3
14:0	15	0.8	4
16:0	17	15	23
16:1 n-7	10.3	1.5	4.5
18:0	7.0	9.1	7.8
18:1	6.2	53	41
18:2 n-6	1	18	12
18:3 n-3	0.5	0.8	-
18:4 n-3	-	-	-
20:1 n-9	-	-	-
20:4 n-6	6	1.6	4.5
20:5 n-3	12	0.2	1
22:5 n-3	7	-	0.3
22:6 n-3	18	-	1.9
			
SFA	39	24.9	34.8
MUFA	16.5	54.5	45.5
N6 PUFA	7	19.6	16.5
N3 PUFA	37.5	1	3.2
N6-N3 ratio	0.2	19.6	5.16

Lipids were extracted from the fish oil (FO), monounsaturated fatty acid rich (MUFA), and N6:N3 diets (% of total fatty acids) as explained under materials and methods. Fatty acid composition was determined by gas liquid chromatography (GLC).

### Plasma Lipid and Lipoprotein Profile

After the four-week feeding period, the animals were fasted for 14 hours prior to sacrifice. The hamsters were anaesthetized by halothane sniffing, heart was punctured and fasting blood samples were collected in tubes containing EDTA (n = 6, F_1_B; n = 6, GS). The remaining animals were used for post-heparin injections for the LPL assay. The plasma was separated by centrifugation at 3000 g for ten minutes and stored on ice at 4°C until further analysis. The F_1_B hamsters fed the fish oil diet had milky plasma containing chylomicron-like particles. These particles were then isolated by centrifugation at 15 500 g for 20 minutes at 12°C. Individual lipoprotein fractions were isolated by sequential density gradient ultracentrifugation on a TL100 fixed angle rotor as described previously [14]. The plasma and individual lipoprotein fractions were analyzed using enzymatic kit methods for total cholesterol (Cholesterol Liquicolor Kit, Stanbio, Texas, USA), TG (Triglyceride Enzymatic Kit, Stanbio Labs, Texas, USA), and free cholesterol (FC) concentrations (Wako Chemicals, Virginia, USA). Cholesterol ester (CE) concentrations were calculated by subtracting free cholesterol concentrations from total cholesterol concentrations. All measurements were performed within one week of plasma collection.

### Hepatic Lipid Profile

Livers were obtained from each hamster, freeze-clamped in liquid nitrogen, and stored at -70°C until further use. Liver lipids were extracted as described previously [16]. The liver lipids were analyzed using enzymatic kit methods for total cholesterol (Cholesterol Liquicolor Kit, Stanbio, Texas, USA), TG (Triglyceride Enzymatic Kit, Stanbio Labs, Texas, USA), and FC (Wako Chemicals, Virginia, USA) concentrations. CE concentrations were calculated by subtracting free cholesterol concentrations from total cholesterol concentrations.

### FPLC Separation of Plasma

Pooled plasma samples (equal volumes from each hamster) were separated by FPLC on a Superose 6 chromatography column (Amersham Biosciences, Quebec, Canada). The plasma cholesterol profile was determined by using an online enzymatic assay reagent (Cholesterol Liquicolor Kit, Stanbio, Texas, USA) as per previously described methods [17].

### Lipoprotein lipase (LPL) activity and LPL mRNA levels

Post-heparin LPL activity was measured using previously described methods [18]. Briefly, after the four-week feeding period and 14 hour fast, F_1_B and GS hamsters (n = 6) on all three diets were anaesthetized and 200 μL of heparin (7500 units/kg body weight) was directly injected into the heart. To allow the LPL enzyme to be released into the plasma, the heparin was allowed to circulate for five minutes. Upon sacrifice, blood was collected by cardiac puncture and separated by centrifugation at 3000 g for ten minutes and stored on ice at 4°C. A separate group of hamsters fed all 3 dietary groups and not injected with heparin were used for pre-heparin LPL activity. LPL activity was assayed using Tri [9, 10-^3^H] oleoyl glycerol (Amersham Biosciences, NJ, USA). A 0.15 M NaCl solution was used in order to measure total lipase activity, while a 3.55 M NaCl solution was used for hepatic lipase activity [18]. LPL activity was calculated by subtracting hepatic lipase activity from total lipase activity, and expressed in nmol/min/ml.

Adipose tissue RNA was extracted using TRIZOL reagent (Invitrogen Life Technologies Inc., Gaithersburg, MD, USA) and LPL mRNA levels were determined by reverse transcription and in vitro DNA amplification. cDNA was synthesized from total liver RNA (2 μg) using Superscript reverse transcriptase (Life Technologies, Burlington, ON, Canada) and used as templates for in vitro DNA amplification reactions. LPL and β-actin mRNA sequences were simultaneously amplified using specific primers. The primer sequence for LPL mRNA amplification was: sense 5'CATTCACCAGAGGGTCACCT3' and anti-sense 5'TTCTTCGTTCAGCAGGGAGT3'. No amplification products were detectable in the absence of reverse transcriptase. The total number of cycles for each PCR reaction was chosen to remain within the exponential phase of the reaction. All PCR reactions were performed in triplicate, and the products were resolved on 1.2% agarose gel. The representative bands were quantitated using gel documentation system. The amount of LPL mRNA was normalized to β-actin mRNA content and expressed as relative units.

### Western blot analysis of Apolipoprotein B (apoB) and Apolipoprotein E (apoE)

The western blot analysis was conducted by the modification of previously published methods [19]. Plasma samples containing 60 μg and 30 μg of protein were used for the immunoblotting of apoB and apoE respectively. Proteins were separated on a 6% (apoB) or 10% (apoE) SDS polyacrylamide gel and transferred to a nitrocellulose membrane. ApoB blots were incubated with goat polyclonal antibody at a dilution of 1:1000 (Calbiochem, California, USA). ApoB was visualized using a secondary bovine anti-goat IgG conjugated to horseradish peroxidase (Santa Cruz, California, USA). ApoE blots were incubated with rabbit polyclonal antibody at a dilution of 1:2000 (Dako, Germany). ApoE was visualized using anti-rabbit-HRP-linked antibody (Calbiochem, California, USA). Both apoB and apoE were detected using an enhanced Luminol chemiluminescence reagent system (Santa Cruz, California, USA). A biotinylated protein ladder (Calbiochem, California, USA) was run with each gel.

### Microsomal triglyceride transfer protein (MTTP) activity assay

Upon sacrifice, the livers and intestine were obtained and freeze-clamped in liquid nitrogen and stored at -70°C. MTTP activity was measured using 150 μg protein from hepatic and intestinal samples from F_1_B and GS hamsters on all three diets using kit methods from Roar Biomedical Inc. (New York, USA). MTTP activity was measured at an excitation wavelength of 465 nm and an emission wavelength of 535 nm. MTTP mediated transfer is observed by the increase in fluorescence intensity as the fluorescent neutral lipid is removed from the self quenched donor to the acceptor. MTTP activity is expressed in nmol/mg/hour.

### Statistical Analysis

The effect of both diet type (D) and animal strain (S) was determined using two-way ANOVA. A Newman-Keuls post-hoc test was used to test significant differences revealed by ANOVA. Values are group means (n = 6, F_1_B; n = 6, GS) and standard error of the mean (SEM). Differences were considered statistically significant when P < 0.05.

## Results

### Plasma Lipid Profile

**Figure 1:**
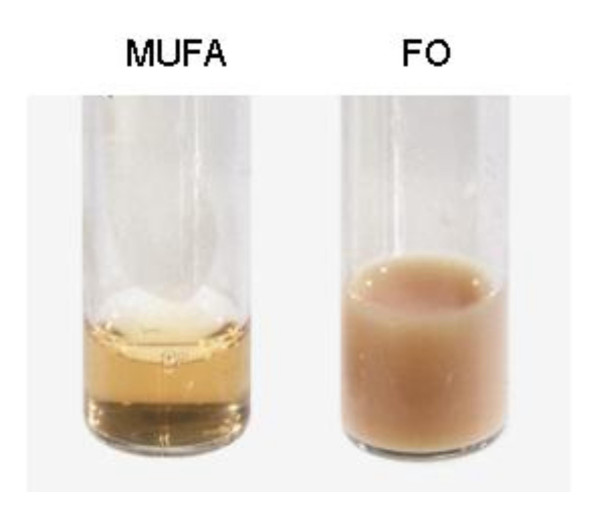
**Fish oil fed F1B hamsters showed milky plasma**. Fasting plasma samples of F_1_B hamsters fed the monounsaturated fatty acid rich (MUFA) and fish oil (FO) diets for four weeks.

**Figure 2:**
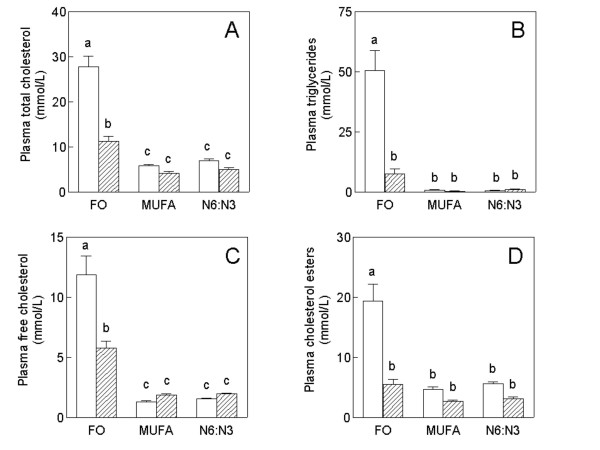
**The plasma lipid profile of F_1_B (solid) and Golden Syrian (GS) (shaded) hamsters**. Hamsters were fed fish oil (FO), monounsaturated fatty acid rich (MUFA), and N6:N3 diets. The specified diets were fed for a period of four weeks. Fasting plasma samples were collected and analyzed for total plasma cholesterol (A), triglycerides (B), free cholesterol (C), and cholesterol esters (D) as described in the material and methods section. Values are means ± SEM (n = 6, F1B; n = 6, GS). Means for a variable with a different letter are significantly different (p = 0.05) by one-way ANOVA, and the Newman-Keuls post-hoc test after a significant interaction between diet and strain was found by two-way ANOVA.

The plasma from fish oil fed F_1_B hamsters was milky and packed with chylomicron-like particles (Figure 1), however milky plasma was not present in fish oil fed GS hamsters. Plasma total cholesterol (A), triglyceride (B), free cholesterol (C), and cholesterol ester (d) concentrations for F_1_B and GS hamsters on all three diets are shown in Figure 2. All lipid parameters were influenced by both diet and animal strain with a significant interaction between the two (P < 0.001). F_1_B hamsters on the fish oil diet had dramatically higher total plasma cholesterol concentrations (P < 0.001), TG concentrations (P < 0.001), FC concentrations (P < 0.001) and CE concentrations (P < 0.001) compared to F_1_B hamsters on the MUFA and n6:n3 diets. Fish oil fed GS hamsters had significantly higher total plasma cholesterol concentrations (P < 0.001) and plasma FC concentrations (P < 0.01) than the GS hamsters on the MUFA and n6:n3 diets. There was no significant effect of fish oil feeding however, on total plasma TG concentration or CE concentration in GS hamsters. While fish oil feeding significantly increased total plasma cholesterol and FC concentrations in both hamster strains, fish oil fed F_1_B hamsters had significantly higher concentrations of all lipid parameters (P < 0.001) than fish oil fed GS hamsters. Plasma cholesterol concentrations in F_1_B hamsters on the fish oil diet were 3 times that of fish oil fed GS hamsters. Similarly, plasma TG concentrations were 5 times greater in fish oil fed F_1_B hamsters when compared to GS hamsters. There was no significant difference between the MUFA and n6:n3 diets for plasma cholesterol, TG, FC, or CE concentrations in either F_1_B or GS hamsters.

**Figure 3:**
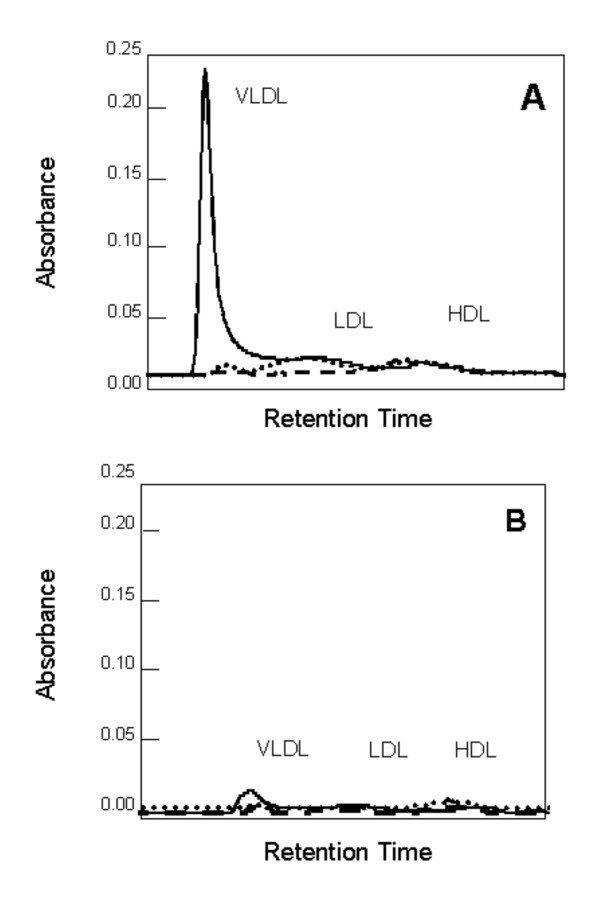
**FPLC profile of fasted plasma samples from F_1_B (A) and Golden Syrian (B) hamsters**. Hamsters were fed the fish oil (FO) (-), monounsaturated fatty acid rich (MUFA) (---), and N6:N3 (⋯) diets. Plasma samples were pooled from six animals in each group, filtered and analyzed on a Superose column as described in the material and methods section.

FPLC profile was carried out to determine the distribution of lipoproteins in fasting plasma samples from both F_1_B and GS hamsters on all three diets (Figure 3). F_1_B hamsters fed the fish oil diet had a significant portion of their plasma cholesterol present in the lower density fractions in comparison to those hamsters on the MUFA and n6:n3 diets (Figure 3A). Fish oil fed GS hamsters also had higher levels of lower density lipoprotein fractions compared to GS hamsters on the MUFA and n6:n3 diets (Figure 3B). This increase however, was dramatically higher for F_1_B hamsters compared to GS hamsters. Both F_1_B and GS hamsters on the MUFA and n6:n3 diets had a comparable amount of their plasma cholesterol in all of the lipoprotein fractions. Due to variations in the lipoprotein profile, individual lipoprotein fractions were separated by ultracentrifugation to measure the concentrations of various lipids.

### Plasma VLDL Profile

There was a marked difference in VLDL composition between F_1_B and GS hamsters (Table 3). VLDL-cholesterol, – TG, and -FC were all influenced by both diet and animal strain, with a significant interaction between the two (P = 0.007, P = 0.0015, and P < 0.0001 respectively). Fish oil feeding to F_1_B hamsters increased VLDL-cholesterol, – TG, -FC, and -CE concentrations in comparison to the MUFA and n6:n3 diets. In GS hamsters, the dramatic effect of fish oil feeding was not as apparent. The VLDL-TG concentrations in fish oil fed F_1_B hamsters in particular, were 25 times that of the GS hamsters. F_1_B and GS hamsters on the n6:n3 diet showed elevated VLDL-cholesterol, -TG, -FC, and -CE compared to respective hamsters strains on the MUFA diet.

**Table 3:** Very-low-density lipoprotein (VLDL) lipid concentrations for F_1_B and Golden Syrian (GS) hamsters

VLDL	Diet	Animal Strain		Statistical Analysis	
(mmol/L)		F1B	GS	Interaction	P value
Cholesterol	FO	8.33 ± 1.57	2.63 ± 0.48	D × S	P = 0.0007
	MUFA	1.20 ± 0.21	0.12 ± 0.04	D	P < 0.0001
	N6:N3	1.47 ± 0.26	0.50 ± 0.17	S	P < 0.0001
					
Triglycerides	FO	25.3 ± 8.82	1.31 ± 0.55	D × S	P = 0.0015
	MUFA	4.11 ± 1.26	0.73 ± 0.25	D	P = 0.0014
	N6:N3	4.28 ± 1.81	1.68 ± 0.31	S	P = 0.0003
					
Free Cholesterol	FO	5.03 ± 1.04	0.12 ± 0.03	D × S	P < 0.0001
	MUFA	0.64 ± 0.21	0.05 ± 0.01	D	P < 0.0001
	N6:N3	1.07 ± 0.63	0.17 ± 0.06	S	P < 0.0001
					
Cholesterol Esters	FO	3.31 ± 0.55	1.32 ± 0.28	D	P < 0.0001
	MUFA	0.57 ± 0.18	0.09 ± 0.03	S	P < 0.0001
	N6:N3	1.91 ± 0.81	0.33 ± 0.12		

Hamsters were fed fish oil (FO), monounsaturated fatty acid rich (MUFA) or N6:N3 diets for 4 weeks. Lipids were analysed as described under materials and methods section. Values are means SEM, (n = 6 for F1B, n = 6 for GS). Groups were compared by two-way ANOVA, p = 0.05. D, S represents diet and strain respectively.

### Plasma LDL Profile

Table 4 shows the LDL lipid profile for F_1_B and GS hamsters. LDL-cholesterol, -TG, and -FC were all independently influenced by both diet (P < 0.02) and animal strain (P < 0.04). Only LDL-TG however showed an interactive effect of both diet and animal strain (P = 0.0118). Fish oil feeding was associated with an increase in LDL-cholesterol, -TG, and -FC concentrations in F_1_B hamsters compared to the F_1_B hamsters fed the MUFA or n6:n3 diet. Fish oil fed GS hamsters also had elevated levels of LDL-cholesterol, -TG, and -FC concentrations compared to those on the MUFA and n6:n3 diets. Comparison of the two strains however, shows that this increase was much more pronounced in F_1_B than GS hamsters. The LDL-cholesterol, TG, and CE concentrations in F_1_B hamsters on the n6:n3 diet were two-fold higher than the F_1_B hamsters fed the MUFA diet. LDL-TG, -FC, and -CE concentrations in GS hamsters were two-fold higher on the n6:n3 diet than GS hamsters fed the MUFA diet.

**Table 4:** Low-density lipoprotein (LDL) lipid concentrations for F_1_B and Golden Syrian (GS) hamsters

LDL	Diet	Animal Strain		Statistical Analysis	
(mmol/L)		F1B	GS	Interaction	P value
Cholesterol	FO	7.18 ± 2.39	2.54 ± 0.10	D	P = 0.0006
	MUFA	1.55 ± 0.19	0.67 ± 0.07	S	P = 0.0004
	N6:N3	3.92 ± 0.56	0.95 ± 0.10		
					
Triglycerides	FO	12.1 ± 4.58	3.06 ± 0.44	D × S	P = 0.0118
	MUFA	0.19 ± 0.05	0.24 ± 0.08	D	P < 0.0001
	N6:N3	0.44 ± 0.23	0.58 ± 0.14	S	P = 0.0348
					
Free Cholesterol	FO	1.71 ± 0.28	1.10 ± 0.04	D	P < 0.0001
	MUFA	0.76 ± 0.24	0.23 ± 0.02	S	P = 0.0005
	N6:N3	0.98 ± 0.36	0.41 ± 0.02		
					
Cholesterol Esters	FO	1.64 ± 0.30	1.10 ± 0.04	D × S	P = 0.0005
	MUFA	1.15 ± 0.26	0.23 ± 0.02	D	P = 0.001
	N6:N3	2.93 ± 0.35	0.41 ± 0.02		P = 0.0003

Hamsters were fed fish oil (FO), monounsaturated fatty acid rich (MUFA) or N6:N3 diets for 4 weeks. Lipids were analysed as described under materials and methods section. Values are means SEM, (n = 6 for F1B, n = 6 for GS). Groups were compared by two-way ANOVA, p = 0.05. D, S represents diet and strain respectively.

### Plasma HDL profile

The analysis of HDL fractions showed a significant effect of strain (P < 0.02) on HDL-cholesterol and -FC concentrations (Table 5). In general, HDL-cholesterol concentrations were higher in F_1_B hamsters than in GS hamsters, but were unaffected by diet. HDL-FC and -TG concentrations were also independently influenced by diet. Fish oil feeding tended to increase HDL-TG and -FC in both F_1_B and GS hamsters compared to the hamsters fed the MUFA and n6:n3 diets. The n6:n3 diet also increased HDL lipid concentrations. Hamsters on the n6:n3 diet had higher HDL-TG concentrations than those on the MUFA diet. N6:N3 feeding resulted in a six-fold increase in HDL-TG in F_1_B hamsters, and a three-fold increase in GS hamsters compared to hamsters fed the MUFA diet.

**Table 5:** High-density lipoprotein (HDL) lipid concentrations for F_1_B and Golden Syrian (GS) hamsters

HDL	Diet	Animal Strain		Statistical Analysis	
(mmol/L)		F1B	GS	Interaction	P value
Cholesterol	FO	1.55 ± 0.30	1.46 ± 0.08	S	P = 0.0163
	MUFA	2.22 ± 0.17	1.46 ± 0.11		
	N6:N3	1.73 ± 0.07	1.53 ± 0.19		
					
Triglycerides	FO	3.23 ± 1.54	0.69 ± 0.12	D	P = 0.0035
	MUFA	0.06 ± 0.01	0.06 ± 0.03		
	N6:N3	0.33 ± 0.12	0.20 ± 0.06		
					
Free Cholesterol	FO	1.24 ± 0.33	0.69 ± 0.07	D	P = 0.0102
	MUFA	0.65 ± 0.22	0.41 ± 0.02	S	P = 0.0120
	N6:N3	0.59 ± 0.20	0.32 ± 0.05		
					
Cholesterol Esters	FO	0.56 ± 0.15	0.94 ± 0.20	D × S	P = 0.0129
	MUFA	1.77 ± 0.25	1.04 ± 0.11	D	P = 0.0035
	N6:N3	1.14 ± 0.20	1.21 ± 0.15		

Hamsters were fed the fish oil (FO), monounsaturated fatty acid rich (MUFA) or N6:N3 diets for 4 weeks. Lipids were analysed as described under materials and methods section. Values are means SEM, (n = 6 for F1B, n = 6 for GS). Groups were compared by two-way ANOVA, p = 0.05. D, S represents diet and strain respectively.

### Hepatic Lipid Profile

Liver is the main site to regulate the metabolism of lipids and cholesterol. Table 6 represents the hepatic lipid concentrations for F_1_B and GS hamsters under all three dietary conditions. Hepatic cholesterol and CE concentrations were independently influenced by diet (P < 0.0001), while there was a significant influence of both diet (P < 0.0001) and strain (P < 0.0001) on hepatic TG concentrations. GS hamsters had higher hepatic TG concentrations than F_1_B hamsters on all three diets. Fish oil feeding however, increased hepatic TG concentrations in both F_1_B and GS hamsters compared to hamsters fed the MUFA and n6:n3 diets. The n6:n3 diet also elevated hepatic lipid concentrations in comparison to the MUFA diet. Liver total cholesterol and CE were approximately 1.5 times higher in F_1_B and GS hamsters on the n6:n3 diet compared to the MUFA diet.

**Table 6:** Hepatic lipid concentrations for F_1_B and Golden Syrian (GS) hamsters

Hepatic lipids	Diet	Animal Strain		Statistical Analysis	
(mg/g liver)		F1B	GS	Interaction	P value
Total Cholesterol	FO	6.86 ± 1.14	7.54 ± 0.43	D	P < 0.0001
	MUFA	3.36 ± 0.29	2.79 ± 0.33		
	N6:N3	5.20 ± 0.89	4.48 ± 0.42		
					
Triglycerides	FO	3.44 ± 0.57	6.13 ± 0.72	D, S	P < 0.0001
	MUFA	1.35 ± 0.35	3.12 ± 0.40		
	N6:N3	1.89 ± 0.29	3.22 ± 0.24		
					
Free Cholesterol	FO	0.65 ± 0.10	0.45 ± 0.02	___	___
	MUFA	0.52 ± 0.08	0.53 ± 0.05		
	N6:N3	0.71 ± 0.13	0.66 ± 0.05		
					
Cholesterol Esters	FO	5.75 ± 0.95	7.10 ± 0.43	D	P < 0.0001
	MUFA	3.01 ± 0.36	2.26 ± 0.41		
	N6:N3	4.50 ± 0.77	3.82 ± 0.43		

Hamsters were fed fish oil (FO), monounsaturated fatty acid rich (MUFA) or N6:N3 diets for 4 weeks. Lipids were analysed as described under materials and methods section. Values are means SEM, (n = 6 for F1B, n = 6 for GS). Groups were compared by two-way ANOVA, p = 0.05. D, S represents diet and strain respectively.

### Apolipoprotein B protein

**Figure 4:**
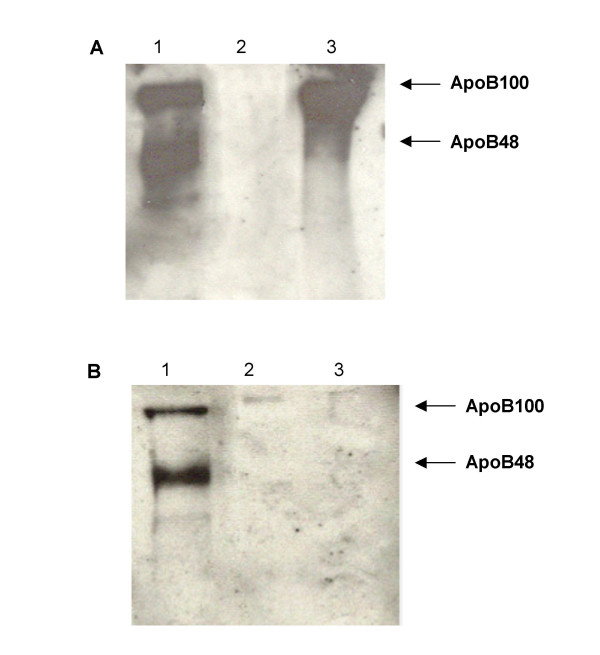
**Apolipoprotein B (apoB) in F_1_B (A) and Golden Syrian (B) hamsters**. Hamsters fed the fish oil, monounsaturated fatty acid rich, and N6:N3 diets are represented in lanes 1, 2, and 3 respectively. Proteins were separated on a 6% SDS polyacrylamide gel and transferred to a nitrocellulose membrane. ApoB proteins were detected by western blotting as described in the materials and methods section.

To determine the origin of various lipoproteins, western blot analysis was performed for apoB48 and apoB100. Figure 4 shows apoB100 and apoB48 in plasma from F_1_B (A) and GS (B) hamsters fed fish oil (lane 1), MUFA (lane 2), and n6:n3 (lane 3) diets. Using 60 μg of total plasma protein, apoB100 was undetectable in F_1_B hamsters fed a MUFA rich diet, however F_1_B hamsters on the fish oil and n6:n3 diets had markedly higher plasma apoB100 protein when 60 μg of plasma total protein was used for the Western blots. Surprisingly, after a 14-hour fast, plasma apoB48 was also very high in F_1_B hamsters fed fish oil diets. Fish oil feeding to GS hamsters had a similar effect as seen in F_1_B hamsters however the effect was not as dramatic. Both apoB100 and apoB48 were higher in fish oil fed GS hamsters, whereas apoB48 was not detectable in the plasma of GS hamsters fed the MUFA or n6:n3 diets. Fish oil fed F_1_B hamsters had markedly higher apoB100 and apoB48 than GS hamsters on the fish oil diet.

### Microsomal triglyceride transfer protein (MTTP) activity

**Figure 5:**
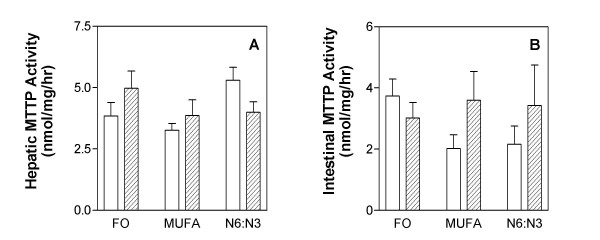
**Hepatic (panel A) and intestinal (panel B) microsomal triglyceride transfer protein (MTTP) activity**. The F_1_B (solid) and Golden Syrian (shaded) hamsters were fed fish oil (FO), monounsaturated fatty acid rich (MUFA) or N6:N3 diets. Animals were fed the specified diets for four weeks. Upon sacrifice, the liver and intestine were removed and snap frozen in liquid nitrogen and stored at -70°. Tissues were then analyzed for MTTP activity as described in the materials and methods section. Means for a variable with a different letter are significantly different (p < 0.05) by two-way ANOVA and the Newman-Keuls post-hoc test.

MTTP plays an important role in the assembly of lipoproteins originating from the liver (VLDL) and the intestine (chylomicrons). Thus, hepatic and intestinal MTTP activity was measured in F_1_B and GS hamsters fed various diets. There was no significant effect of diet (P = 0.15) or strain (P = 0.77) on hepatic MTTP activity (Figure 5A). Similarly, there was no significant effect of diet (P = 0.74) or strain (P = 0.31) on intestinal MTTP activity (Figure 5B). We also measured MTTP mRNA and protein levels however there was no significant effect of diet (data not given).

### Lipoprotein lipase (LPL) activity and mRNA levels

**Figure 6:**
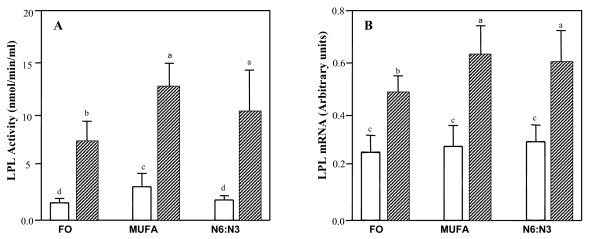
**Lipoprotein lipase (LPL) activity (panel A) and LPL mRNA levels (panel B)**. The F_1_B (solid) and Golden Syrian (shaded) hamsters were fed fish oil (FO), monounsaturated fatty acid rich (MUFA), and N6:N3 diets. The specified diets were fed for a period of four weeks. After a 14-hour fast, heparin was injected directly into the heart; blood was collected and assayed for LPL activity (panel A) as described in the materials and methods section. Total RNA was extracted from adipose tissue and LPL mRNA levels were measured (panel B) using specific primers as described under the materials and methods section. Means for a variable with a different letter are significantly different (p < 0.05) by two-way ANOVA and the Newman-Keuls post-hoc test.

Lipoprotein lipase plays a significant role in the hydrolysis of VLDL and chylomicrons. Since the plasma from F_1_B hamsters fed fish oil was milky and also contained very high levels of VLDL, the activity of LPL was measured in F_1_B and GS hamsters fed various diets (Figure 6A). There was a significant effect of strain (P < 0.001) on lipoprotein lipase activity, where F_1_B hamsters had markedly lower post-heparin LPL activity compared to GS hamsters on all three diets. Both fish oil and n6:n3 feeding reduced LPL activity in F_1_B hamsters, where fish oil feeding had a greater effect. Fish oil fed GS hamsters also showed reduced LPL activity. There was, however no effect of diet or hamster strain on pre-heparin LPL activity (data not shown). We also measured LPL mRNA levels in adipose tissue of F_1_B and GS hamsters fed various diets (Figure 6B). The F_1_B hamsters showed significantly lower LPL mRNA levels as compared to the GS hamsters. There was no effect of diet on LPL mRNA levels in F_1_B hamsters, however fish oil feeding reduced LPL mRNA levels in GS hamsters as compared to GS hamsters fed MUFA or n6:n3 diet.

### Plasma apolipoprotein E protein

**Figure 7:**
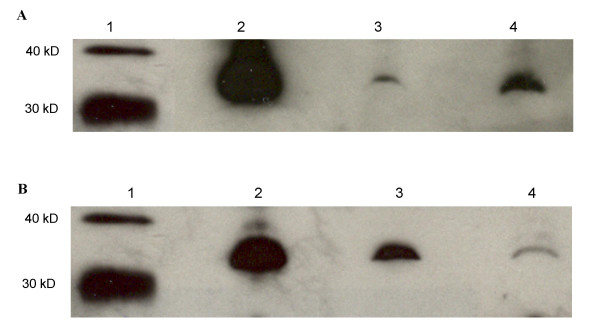
**Apolipoprotein E (apoE) in F_1_B (A) and Golden Syrian (B)**. Lane 1 shows the molecular weight marker. Hamsters fed the fish oil, monounsaturated fatty acid rich, and N6:N3 diets are represented in lanes 2, 3, and 4 respectively. Proteins were separated on a 10% SDS polyacrylamide gel and transferred to a nitrocellulose membrane. ApoE proteins were detected by western blotting as described in the materials and methods section.

Figure 7 shows plasma apoE in F_1_B (A) and GS (B) hamsters on the fish oil (lane 2), MUFA (lane 3), and n6:n3 (lane 4) diets. All three diets had varying effects on plasma apoE in F_1_B and GS hamsters. F_1_B hamsters on the fish oil diet had markedly higher plasma apoE than F_1_B hamsters on the MUFA and n6:n3 diets. F_1_B hamsters on the n6:n3 diet had much higher plasma apoE than those on the MUFA diet. The levels of plasma apoE in GS hamsters in response to fish oil feeding were similar to that seen in the F_1_B hamsters. Fish oil fed GS hamsters had markedly higher plasma apoE than GS hamsters on the MUFA and n6:n3 diets. In addition, GS hamsters on the MUFA diet had higher apoE compared to those hamsters on the n6:n3 diet.

There is an apparent difference in apoE between F_1_B and GS hamsters on all three diets. F_1_B hamsters on the fish oil diet showed an increase in apoE compared to fish oil fed GS hamsters. In addition, the effects of the MUFA and n6:n3 diets in F_1_B hamsters are reversed in GS hamsters. MUFA fed F_1_B hamsters show a decrease in apoE compared to MUFA fed GS hamsters, while F_1_B hamsters on the n6:n3 diet have markedly higher apoE than GS hamsters on the n6:n3 diets.

## Discussion

Fish oil has typically been shown to reduce plasma TG concentrations and to promote the presence of a healthy lipoprotein profile. However there are controversial reports on the effect of fish oil on plasma total cholesterol and LDL-cholesterol levels. Since the plasma lipoprotein profile of hamsters is similar to human lipoprotein profile, we used hamster as an animal model to investigate the regulation of plasma cholesterol levels by dietary fish oil. The F_1_B hamster is a hybrid strain that develops severe hyperlipidaemia and atherosclerosis at much lower dietary cholesterol concentrations compared to the GS hamsters [20]. However the mechanisms behind severe hyperlipidaemia in F_1_B hamsters are unknown. Our previous findings have shown that increasing the quantity of fat in the diet, especially from fish oil, caused severe hyperlipidaemia in F_1_B hamsters [14]. To clarify the controversies associated with fish oil mediated regulation of plasma cholesterol levels we used high fat fish oil diet and compared the inbred F_1_B hamsters to the normal outbred GS hamsters for alterations in lipoprotein metabolism. Higher than normal levels of fish oil were used to observe an exaggerated effect of fish oil on plasma lipid and lipoprotein levels to be able to understand the mechanisms involved. The response of F_1_B and GS hamsters to various diets was different where F_1_B hamsters showed severe hyperlipidaemia when fed a high fat fish oil diet compared to GS hamsters, highlighting the possibility of heterogeneity between genetic background [20,21]. GS hamsters typically have increases in both LDL- and HDL-cholesterol in response to an atherogenic diet [22]. In contrast, F_1_B hamsters have highly elevated LDL-cholesterol concentrations without appreciable effects on HDL-cholesterol, further suggesting genotypic differences between hamster strains [20].

Differences in dietary response were apparent upon separation of the plasma from F_1_B and GS hamsters. F_1_B hamsters on the high fat fish oil diet had milky plasma, packed with chylomicron-like particles even after subjection to a 14-hour fast. This effect was not observed in GS hamsters on the fish oil diet. Fish oil supplementation has typically been shown to decrease apoB protein, and VLDL secretion from hamster hepatocytes [7], a quality that is characteristic of the hypotriglyceridaemic properties of fish oil. However, fish oil can significantly elevate apoB levels in hypertriglyceridaemic subjects [23].

In addition to milky plasma, F_1_B hamsters on the fish oil diet had markedly higher plasma cholesterol, TG, FC, and CE concentrations in comparison to GS hamsters, which are consistent with other studies [18,20,21,24,25]. The response of these two strains to a diet rich in n6:n3 was also different. We noted an increase in LDL-cholesterol concentrations in F_1_B hamsters on the n6:n3 diet than LDL-cholesterol concentrations in the GS hamster. Previously we observed that fish oil feeding to F1B hamsters inhibited the mRNA expression of LDL-receptor [14]. We did not measure the expression of LDL-receptor in the current study however it is likely that n-3 fatty acids inhibited LDL-receptor expression under the current dietary conditions in F_1_B hamsters causing an increase in plasma LDL levels. Fish oil feeding to both F1B and GS hamsters showed an increase in HDL-triglyceride concentrations as compared to hamsters fed diets rich in n6:n3 or MUFA. There was however no significant change in HDL-cholesterol concentrations. An increase in HDL-triglycerides might suggest enhanced clearance of HDL in the fish oil fed hamsters, which deserves future investigations. The hepatic lipid profile also showed a significant increase in triglyceride levels in fish oil fed F1B hamsters, however the increase was not as dramatic compared to plasma. This could be due to several factors, i.e. lipids not delivered to the liver, low lipoprotein lipase activity and/or inhibition of hepatic triglyceride synthesis.

The FPLC lipoprotein profile indicates that different responses to a high fat fish oil diet occur between the two hamster strains, where fish oil fed F_1_B hamsters have a significant portion of their plasma cholesterol in the lower density lipoproteins compared to GS hamsters. In an attempt to locate the lipoprotein source of the over-abundance of plasma TG, a western blot analysis for apoB48 and apoB100 was conducted. The detection of apoB48 and apoB100 indicate the presence of intestinally and hepatically derived lipoproteins respectively. We observed that F_1_B and GS hamsters on fish oil diet had markedly higher plasma apoB48 and apoB100 protein than hamsters on the MUFA and n6:n3 diets. Fish oil has been shown to decrease apoB secretion and suppress apoB protein expression specifically by increasing the intracellular degradation of apoB [7,26]. However, there are reports showing an increase in apoB concentrations HepG2 cells treated with fish oil [27]. Furthermore, it has been shown that the presence of apoB48 in F_1_B hamsters on a high fat coconut diet is responsible for elevated plasma total cholesterol and TG concentrations [18].

In addition to dietary induced variation in apoB protein, the effect of hamster strain is also apparent. While apoB48 and apoB100 were elevated in both F_1_B and GS hamsters on the fish oil diet, this effect was more obvious in F_1_B hamsters. Similarly, apoB100 was elevated in F_1_B hamsters on the n6:n3 diet, but not in GS hamsters on the same diet. The apoB protein levels parallels their differences in plasma lipid and lipoprotein concentrations, where F_1_B hamsters had significantly greater concentrations of apoB-containing lipoproteins than GS hamsters. There are two plausible explanations for the variation in apoB: a) enhanced synthesis and secretion of apoB-containing lipoproteins, or b) a decrease in the clearance of these lipoproteins from the plasma.

We investigated the effects of diet and strain on MTTP activity, which is involved in the formation of VLDL and chylomicrons from the liver and intestine respectively. MTTP is involved in the synthesis and secretion of TG-rich lipoproteins, and transfers lipid from the endoplasmic reticulum membrane to apoB48 or apoB100 for the subsequent formation of chylomicrons or VLDL respectively. We expected to see an increase in MTTP activity in hamsters on the fish oil diet; however no effects of dietary fat or hamster strain were observed. The lack of effect of dietary fat or animal strain on both hepatic and intestinal MTTP activity, in addition to the abundance of evidence suggesting that fish oils decrease VLDL-secretion, indicate that it is not the secretion of TG-rich lipoproteins that is affected, but the clearance of these lipoproteins and their remnants from the plasma.

LPL is the rate-limiting determinant for the hydrolysis of chylomicrons and VLDL within the plasma. The presence of apoB48 in F_1_B and GS hamsters after an extended fast indicate the presence of chylomicrons in the plasma, in addition to severe postprandial dyslipidemia in fish oil fed hamsters. We found a significant effect of animal strain and diet on post-heparin LPL activity. F_1_B hamsters had significantly lower post-heparin LPL activity than GS hamsters, further reiterating variation in genetic background between hamster strain. Similar results have been found in F_1_B hamsters, where this strain had significantly lower post-heparin LPL activity compared to DSNI hamsters [18]. This response however, was dependent on dietary cholesterol intake. Previous reports using other animal models has shown that n-3 fatty acids do not significantly influence post-heparin LPL [10-13,28-31]. However, we observed a significant decrease in LPL activity on a high fat fish oil diet in F_1_B hamsters. Thus, it is likely that fish oil mediated hyperlipidaemic effect is partially due to a decrease in LPL activity. The F_1_B hamsters also showed significantly lower adipocyte LPL mRNA levels as compared to the GS hamsters. LPL mutations are found in human populations at a frequency of 1:1000 [32].

LPL deficiency leads to hyperlipidaemia, which is characterized by the pathological presence of chylomicrons, together with elevated VLDL after a 12–14 hour fast similar to what we observed in F1B hamsters. A decrease in LPL activity however should cause a decrease in plasma LDL levels due to inhibition of VLDL conversion to LDL. We observed an increase in plasma LDL concentrations in fish oil fed F_1_B hamsters. This is likely due to inhibition of LDL-receptor gene expression by fish oil in F_1_B hamsters [14].

The presence of apolipoprotein E is another factor that has a critical role in the hepatic clearance of TG-rich lipoproteins from the plasma. In an apoE knockout mouse for example, severe hypertriglyceridaemia similar to our phenotype, was seen when these mice were fed a high fat fish oil diet [33]. It was determined by these authors that apoE was required for fish oil to exert its hypotriglyceridaemic effects. In the present study however, F_1_B and GS hamsters on the fish oil diet had dramatically higher apoE than those hamsters on the MUFA and N6:N3 diets. In addition, the presence of apoE was much greater in fish oil fed F_1_B hamsters than GS hamsters further highlighting strain-specific differences in these hamsters. While we are the first to demonstrate this effect in hamsters, other investigators have previously shown that the overexpression of apoE can also induce hypertriglyceridaemia [34]. Overexpression of human apoE in the rabbit at increasing concentrations of apoE results in a net increase in circulating TG levels [34]. While plasma apoE concentrations were not established in this study, it can be speculated that higher levels of apoE in fish oil fed hamsters may partially contribute to the observed hyperlipidaemia. Moreover, it has been shown that elevated levels of apoE can inhibit LPL-mediated hydrolysis of TG-rich lipoproteins [35]. Thus the elevated apoE protein expression in fish oil fed F_1_B hamsters may partially explain the decline in LPL activity in this strain.

## Conclusion

The comparison of the inbred F_1_B hamster to the outbred, normal GS hamsters, allowed us to attribute fish oil induced hyperlipidaemia to diversity in animal strain. The F_1_B hamsters show a dramatic hyperlipidaemic response to fish oil compared to GS hamsters. The observed dyslipidaemia induced by fish oil was a result of the hindrance of TG-rich lipoprotein clearance. Decreased LPL concentrations in F_1_B hamsters elude to heterogeneity within genetic background that contributes to the varied response of these hamsters to dietary fat. Investigation into the regulation of lipoprotein metabolism in these strains will provide insight into the nutrient-gene relationship and the controversial effects of dietary fats on cardiovascular health. Further, identification of polymorphisms within each animal strain in response to various unsaturated fats may provide insight into the diverse and controversial effects of fish oil on cholesterol and lipoprotein metabolism in humans.

## Abbreviations

ApoB, apolipoprotein B; apoE, apolipoprotein E; CVD, cardiovascular disease; CE, cholesterol esters; D, diet type; FC, free cholesterol; GS, Golden Syrian; HDL, high-density lipoprotein; LDL, low-density lipoprotein; LPL, lipoprotein lipase; MTTP, microsomal triglyceride transfer protein; MUFA, monounsaturated fatty acids; N6:N3, diet with an omega-6 to omega-3 ratio of 5; S, animal strain; SFA, saturated fatty acids; TG, triglycerides; VLDL, very-low density lipoprotein.

## Competing interests

The author(s) declare that they have no competing interests.

## Authors' contributions

SKC designed the study, provided interpretation of data and drafted the final manuscript. MLC made contributions to acquisition of data and analysis.
